# Sickle cell nephropathy with diffuse proliferative lupus nephritis: a case report

**DOI:** 10.1186/1746-1596-3-9

**Published:** 2008-02-28

**Authors:** Kamal V Kanodia, Aruna V Vanikar, Kamal R Goplani, Sonia B Gupta, Hargovind L Trivedi

**Affiliations:** 1 Department of Pathology, Laboratory Medicine and Transfusion Services, and Immunohematology, G.R. Doshi and K.M. Mehta Institute of Kidney Diseases and Research Centre and Dr. H. L. Trivedi Institute of Transplantation Sciences, Civil Hospital Campus, Asarwa, Ahmedabad, India; 2 Department of Nephrology and Transplantation Medicine, G.R. Doshi and K.M. Mehta Institute of Kidney Diseases and Research Centre and Dr. H. L. Trivedi Institute of Transplantation Sciences, Civil Hospital Campus, Asarwa, Ahmedabad, India

## Abstract

**Background:**

Sickle cell nephropathy (SCN) is an important cause of mortality in patients with sickle cell disease. SCA with systemic lupus erythematosus (SLE) is known in children and less common in adults, however diffuse proliferative lupus nephritis (DPLN) with SCN has rarely been reported in adults. It requires early diagnosis and aggressive management.

**Case presentation:**

We present here a 35 years old lady with sickle cell disease who presented with edema, dyspnoea on exertion, pyuria and had raised s. creatinine of 7 mg%. Her biopsy revealed SCN with DPLN. She is on maintenance hemodialysis after 2 months of diagnosis.

**Conclusion:**

DPLN with SCN is a rare entity with poor prognosis, which may be overlooked and needs aggressive management.

## Introduction

Sickle cell nephropathy (SCN) is an important cause of mortality in patients with sickle cell disease (SCD) [1]. SCN includes hematuria, papillary necrosis, urinary concentrating defect, impaired renal acidification and potassium excretion, supranormal proximal tubular function, proteinuria, and renal failure^1^. Incidence of renal failure in SCD ranges from 5 to 18% [2]. SCN associated with diffuse proliferative lupus nephritis (DPLN) is a rare entity. We present here a 35 year old lady with sickle cell disease (SCD) and DPLN.

## Case presentation

A 35 year old female with SCD, presented with pedal and periorbital edema, distension of abdomen, decreased urine output and dyspnoea on exertion since 1 month. On examination, she was pale, with pulse rate of 100/minute, normal temperature, and blood pressure was 160/100 mm Hg. Her abdomen was distended due to moderate ascites however there was no organomegaly/scars. Cardio-respiratory and neurological examination was unremarkable. Ultrasonography showed right kidney, 10.9 × 6.0 cm, left kidney of 11.4 × 5.8 cm, with increased echogenicity and maintained cortico-medullary differentiation. Moderate ascites was present.

On investigations, urine albumin was 500 mg/24 hours, microscopy showed 30–40 pus cells and 3–5 granular casts/high power field, urine culture was sterile, serum creatinine, 7.0 mg%, serum proteins, 4.5 gm/dL; serum albumin, 1.8 gm%, serum bilirubin was 0.5 mg/dL, serum alanine amino transferase was 16 units/L, random blood sugar was 87 mg/dL, serum uric acid was 7.5 mg/dL, serum sodium was 134 meq/L and serum potassium, 5.5 meq/L and positive sickling test was noted at 24 hours. Her hemoglobin was 7.1 gm/dL; total leucocyte count was 7,700/cmm with differential count showing neutrophils, 76%, lymphocytes, 20% and eosinophils and monocytes, each, 2%. Peripheral smear showed few crenated and sickled cells with mild hypochromia and anisopoikylocytosis.

**Figure 1:**
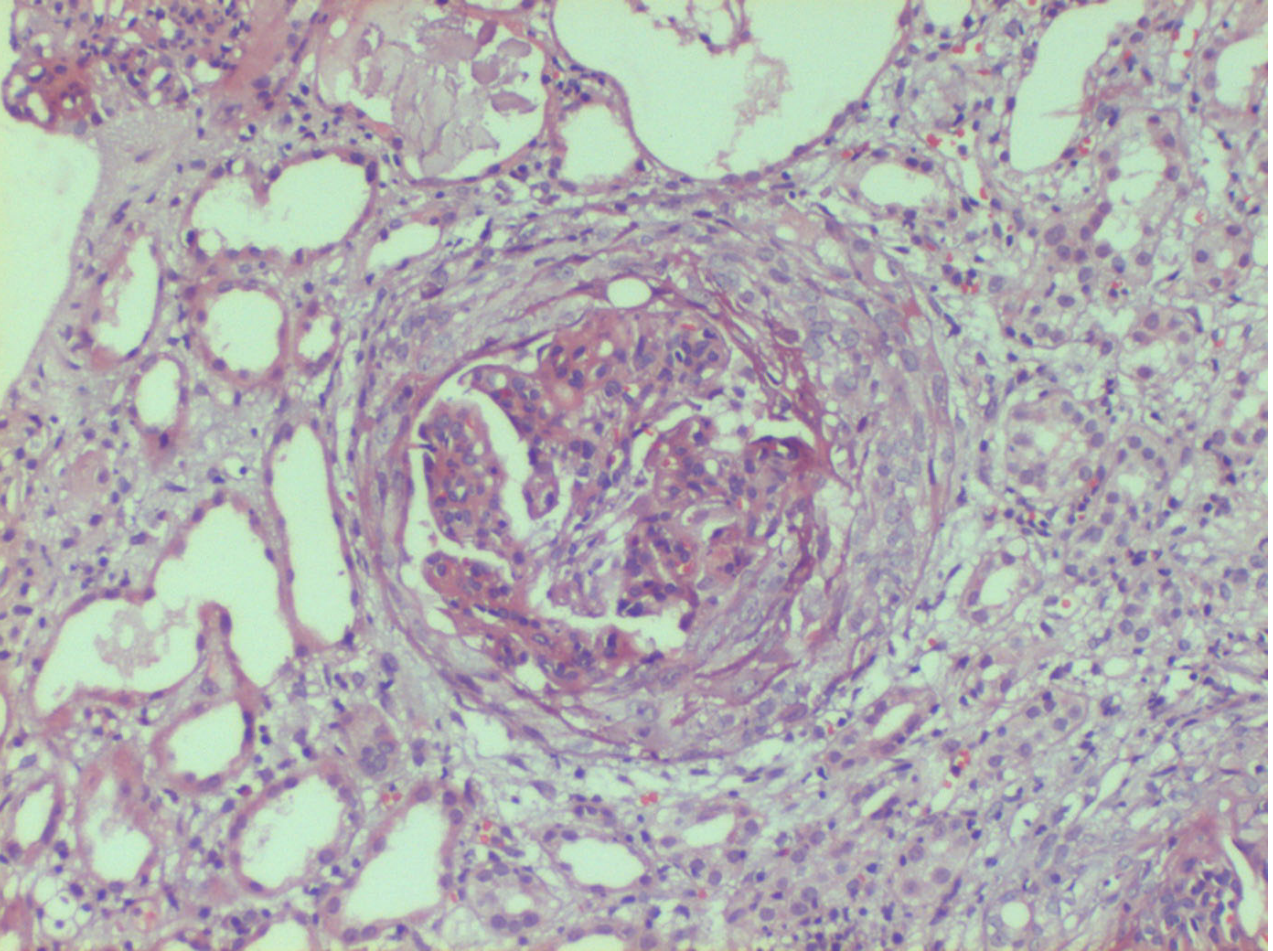
One glomerulus with circumferential cellular crescent and diffuse endocapillary proliferation infiltrated with few leucocytes and RBCs, and surrounded by focally atrophied or moderately degenerated tubules. Diffuse leucocytic infiltration in parenchyma noted; Hematoxylin and eosin stain, × 100.

**Figure 2:**
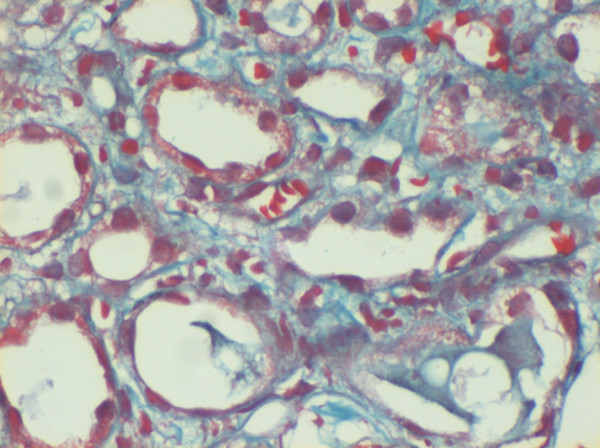
Dilated peri-tubular capillaries filled with sickled RBCs, original Gomori's trichrome stain, × 400.

She was dialyzed and 2 units of packed cells were transfused. Renal biopsy was performed and after paraffin embedding, 3 micrometer sections were taken and stained using Hematoxylin and eosin, Periodic Acid Schiff, Jone's silver methaneamine and Gomori's trichrome stains. Indirect immunofluorescence studies were performed using anti-human IgG, IgM, IgA, C1q, C3, albumin and fibrinogen anti-sera (DAKO, USA). Histopathology revealed one core with 13 glomeruli. All of them were moderately enlarged in size and 11 had exuberant and occasionally circumferential cellular (rarely fibrocellular) crescents occupying 70–90% urinary spaces, occasionally replacing capillary tufts and shrinking or pushing the capillary tufts towards vascular poles (Figure 1). Capillary tufts had fairly open lumina lined by membranes with occasional reduplication, thickening/rupture. There was uniform moderate mesangial prominence. Capillaries and crescents were infiltrated by few sickle shaped, dysmorphic RBCs, platelet/fibrin thrombi. Afferent and efferent arterioles were filled with sickled RBCs. Bowman capsules were segmentally thickened and occasionally ruptured evoking periglomerular leucocytic reaction. Tubules were moderately degenerated and filled with cellular casts and rarely RBCs. Focal atrophy was also evident. Interstitium was moderately prominent for focal fibrosis and overlying focal mononuclear cellular infiltration with admixed neutrophils. Peri-tubular capillaries were dilated and filled with sickled RBCs (Figure 2). At least one medium caliber and two small caliber arteries showed lumina filled with trapped dysmorphic RBCs.

**Figure 3:**
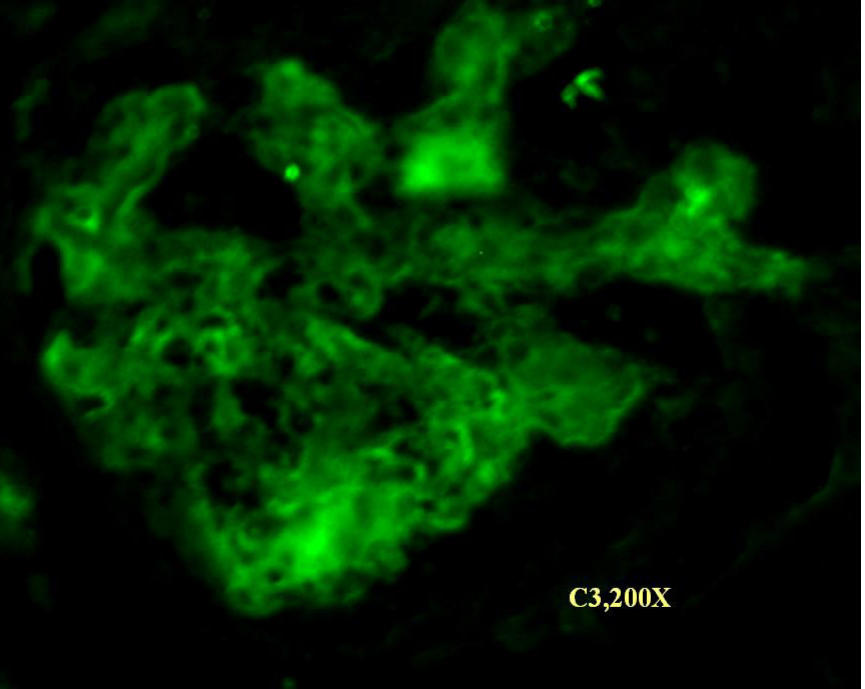
Immunofluorescence study showing anti-human C3 deposition in mesangium and occasional capillaries of glomerulus.

Immunofluorescence studies showed fine granular fluorescence (+2) across 60–80% mesangial regions and adjacent capillaries of all glomeruli on staining with anti-human C3 and (trace/+1) with anti-human IgG, C1q and fibrinogen antisera (Figure 3). Subsequently she was investigated for vasculitis and lupus nephritis. Indirect immunofluorescence and ELISA revealed anti-nuclear antibodies, (normal index: <1, test: 8.5), anti-dsDNA antibodies were >200 IU/mL (normal <25 IU/mL, Orgentec, Germany). Anti-neutrophil cytoplasmic antibodies were absent. Final diagnosis was made as SCN with DPLN, ISN/RPS class IV-G (A).

**Figure 4:**
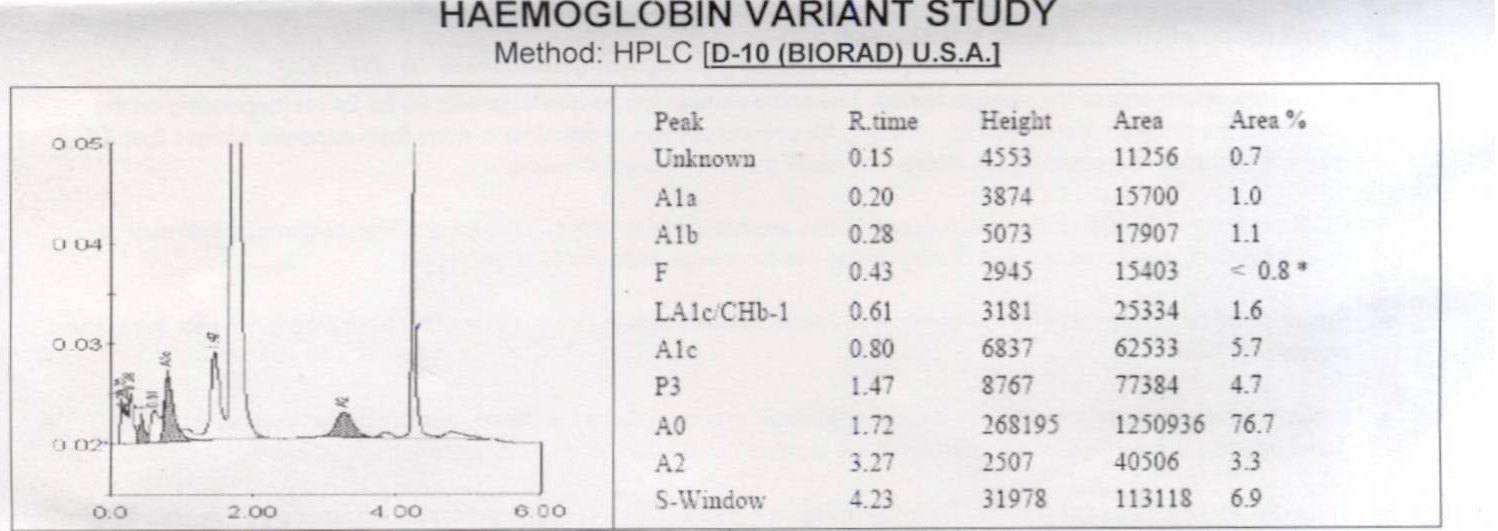
Haemoglobin variant study suggestive of HbSA.

Hemoglobin variant study using High Performance Liquid Chromatography (d-10, Biorad, USA) was performed 5 days after transfusion which showed HbSA of 6.9% (Figure 4).

She was treated with Methylprednisolone 500 mg, intravenously for 3 days and Cyclophosphamide, 500 mg intravenously immediately and dose was repeated after 21 days. Then she was switched over to oral Prednisolone, 40 mg/day. Hemodialysis was done on alternate days for 21 days. At follow-up of 2 months, she is on maintenance dialysis with urine output of less than 400 ml/day.

## Discussion

Patients with SCA are known to develop idiopathic nephrotic syndrome with focal and segmental glomerulosclerosis or with membranoproliferative glomerulonephritis. Systemic lupus erythematosus (SLE) has been reported in adults and more frequently in children with SCD [3,4]. However lupus nephritis with SCN has been rarely documented in adults. It is postulated that a defect in the alternate pathway of complement activity seen in patients with SCD may predispose to immune complex disorders [5]. There are broad spectrum of systemic complications associated with SCD and/or SLE [6,7]. Mesangial expansion, basement membrane duplication as well as occurrence of immune complex disorders in SCD may delay the unmasking of lesion due to SLE unless serological work-up is done. This is a rare case of DPLN along with SCN in an adult, who has become dialysis dependent.

## Conclusion

SCN with DPLN is a rare clinico-pathologic entity which requires timely diagnosis and aggressive management.

## Abbreviations

DPLN: Diffuse Proliferative Lupus Nephritis

SCD: Sickle Cell Disease

SCN: Sickle cell nephropathy

SLE: Systemic lupus erythematosus

## Competing interests

The author(s) declare that they have no competing interests.

## Authors' contributions

KK and AV have made the diagnosis by taking care of laboratory management, histopathology, and drafting the manuscript. KG, SG and HT are involved in the clinical management of this patient. HT also finalized the manuscript. All the authors read and approved the manuscript.
